# The transcriptome of rat hippocampal subfields

**DOI:** 10.1016/j.ibneur.2022.09.009

**Published:** 2022-10-03

**Authors:** João P.D. Machado, Maria C.P. Athie, Alexandre H.B. Matos, Iscia Lopes-Cendes, André.S. Vieira

**Affiliations:** a Department of Structural and Functional Biology, Institute of Biology, University of Campinas (UNICAMP), Campinas, São Paulo, Brazil; b Department of Translational Medicine, School of Medical Sciences. University of Campinas (UNICAMP), Campinas, São Paulo, Brazil; c Brazilian Institute of Neuroscience and Neurotechnology (BRAINN), Campinas, São Paulo, Brazil

**Keywords:** Hippocampus, RNA-Seq, Transcriptomics, Laser-capture microdissection, Hippocampal subfields

## Abstract

The hippocampus comprises several neuronal populations such as CA1, CA2, CA3, and the dentate gyrus (DG), which present different neuronal origins, morphologies, and molecular mechanisms. Laser capture microdissection (LCM) allows selectively collecting samples from target regions and eliminating unwanted cells to obtain more specific results. LCM of hippocampus neuronal populations coupĺed with RNA-seq analysis has the potential to allow the exploration of the molecular machinery unique to each of these subfields. Previous RNA-seq investigation has already provided a molecular blueprint of the hippocampus, however, there is no RNA-seq data specific for each of the rat hippocampal regions. Serial tissue sections covering the hippocampus were produced from frozen brains of adult male Wistar rats, and the hippocampal subfields CA1, CA2, CA3, and DG were identified and isolated by LCM. We found evident segregation of the transcriptomic profile from different regions of the hippocampus and the expression of known, as well as novel, specific marker genes for each region. Gene ontology enrichment analysis of CA1 subfield indicates an enrichment of actin regulation and postsynaptic membrane AMPA receptors genes indispensable for long-term potentiation. CA2 and CA3 transcripts were found associated with the increased metabolic processes. DG expression was enriched for ribosome and spliceosome, both required for protein synthesis and maintenance of cell life. The present findings contribute to a deeper understanding of the differences in the molecular machinery expressed by the rat hippocampal neuronal populations, further exploring underlying mechanisms responsible for each subflied specific functions.

## 1 Introduction

The Hippocampus is one of the most studied structures of the nervous system. It is involved in diverse functions such as spatial navigation, processing of memories and emotional responses, and presents subregions or subfields arranged in a complex circuit (Witter et al., 2014). Each of these subfields, CA1, CA2, CA3, and the dentate gyrus (DG), have different neuronal origins, morphologies, and molecular mechanisms (Hayashi et al., 2015). CA1, CA2 and CA3 subfields are mainly composed of pyramidal cells, creating an output circuitry for the hippocampus. On the other hand, DG cells are the input sub-region, consisting mainly of granule cells (Hainmueller and Bartos 2020). All these cells are in constant communication with different types of inhibitory neurons, which control the excitatory waves produced on the hippocampus (Albrecht et al., 2020, Booker and Vida, 2019). Therefore, an accurate selection of cell populations would preserve regional characteristics, potentially helping to better understand differences in the hippocampal functionality.

Laser capture microdissection (LCM) has enabled an accurate microstructure isolation using a laser coupled to a microscope, which cuts accordingly to a trajectory predefined by the user (Espina et al., 2006). LCM technique allows selectively collecting samples from target regions and eliminating unwanted cells to obtain more specific results (Datta et al., 2015). The LCM process does not alter the integrity of a collected sample, thus it is an excellent method to collect cells or cell subfields preserving RNA integrity (Espina et al., 2006). The use of regional LCM to isolate CA1, CA2 and CA3 pyramidal layers, and DG granular layer would allow the exploration of the molecular machinery unique to each of these subfields. In conjunction with transcriptomic tools, it is possible to define expression characteristics of a given neuronal population and to obtain region-specific transcriptomes, essential for a deeper insight into hippocampus function.

Despite all hippocampal transcriptome investigations done so far, most have not explored the regional heterogeneities of this structure. Furthermore, gene expression features such as gene ontology and quantitative analysis of transcripts remain unclear and need further investigation. Studies separating the hippocampal subfields have been essentially performed by microarrays (Greene et al., 2009, Lein et al., 2004, Masser et al., 2014, Nakamura et al., 2011; Zhao et al., 2001). Previous RNA-seq investigation has already provided a molecular blueprint of the mouse hippocampus (Cembrowski et al., 2016, Farris et al., 2019) and rat hippocampus (Smith et al., 2020), however there is no RNA-seq data for the specific laser capture microdissection of the rat hippocampal subfields.

Here, we explore the gene expression heterogeneity of rat hippocampus by using LCM to isolate its different subfields (CA1, CA2, CA3, DG), and by tracing a profile of those regions transcriptome. We also provide insights into the transcriptional organization of specific enriched ontologies or pathways for each subfield and interpret possible functional characteristics associated with such expression patterns.

## 2 Methods

### 2.1 Animals and laser microdissection (LCM)

In this study, three month old male Wistar rats (n = 4) were housed in a ventilated environment (12 h/12 h light cycle) with ad libitum access to standard rodent chow and water. All procedures were executed according to the ethical standards for animal experimentation at the University of Campinas-UNICAMP (Brazilian federal law 11.794 (10/08/2008 - Animal Use Ethics Committee protocol 2903–1). The statistical calculation of sample size was carried out using the RNASeqPower package (https://bioconductor.org/packages/release/bioc/html/RNASeqPower.html) in R environment. Rats were anesthetized with isoflurane (2% isoflurane, 98% oxygen at 1 liter/min) and decapitated using a small animal guillotine. Then, brains were snap-frozen at − 55 °C and posteriorly processed in a cryostat (Leica Biosystems - Wetzlar, Germany) to obtain 40-µm serial sections covering the entire hippocampus. A total of 120 coronal sections were produced covering the entire hippocampus based on Paxinos Rat Brain Atlas 7th edition coordinates (Bregman −1.72 mm AP to −6.72 mm AP) and all 120 sections were used for hippocampus LCM. For tissue section processing (Fig. S1), the LCM system manufacturer guidelines for RNA handling (Carl Zeis PALM protocols - RNA handling document) were used, briefly, these were immediately collected in PEN membrane-covered slides (Life Technologies®, Thermo Fisher Scientific - Waltham, USA) and stained with Cresyl Violet, dehydrated with an ethanol series and stored at − 80 °C.

For laser microdissection, the hippocampal subfields were identified according to Paxinos Rat Brain Atlas 7th edition (Paxinos & Watson, 2013) and delimited with a Palm (Zeiss® - Jena, Germany) system. We followed the hippocampal microdissection methods outlined by Vieira et al. (2016), concerning the hippocampal subfields. Finally, tissue was mechanically collected in separate microcentrifuge tubes using a surgical microscope and micro-forceps. We collected the granular subfield of the DG and the pyramidal subfields of CA1, CA2 and CA3. The CA2 subfield was distinguished by pyramidal cells similarity to those in CA3 than CA1 but with more compact grouping of neurons than CA3.

### 2.2 Library preparation and Next-generation sequencing

Samples RNA was extracted with TRIzol (Thermo Fisher Scientific - USA) using the manufacturer instructions. Recovered RNA from all samples presented an average integrity quality number (RIN) of 7 as verified by 2100 Bioanalyzer Instrument using Agilent RNA 6000 Pico kit (Agilent, CA, USA). Then, cDNA libraries were reverse-transcribed from 200 ng of extracted RNA using TruSeq Stranded Total RNA LT (Illumina®, CA, USA), according to the manufacturer instructions. Eight barcoded libraries were pooled per lane to be sequenced in a HiSeq® 2500 (Illumina®, CA, USA) in High Output mode, producing 100-bp paired-end sequences. A total of 253,341,913 100-bp paired-end reads were produced from all samples, averaging 15.7 million paired-end reads per sample. The average sequence alignment was 77,5% and read counts per gene were used to estimate gene expression and statistical analysis in DESEQ2. The datasets generated here were deposited in the National Center for Biotechnology Information (NCBI) Gene Expression Omnibus (GEO), accession number GSE179101.

### 2.3 Data processing and differentially expressed genes (DEGs)

All sequenced reads were aligned using the StarAligner 2.6 program (https://github.com/alexdobin/STAR, RRID:SCR_004463) (Dobin et al., 2013) with the Rattus norvegicus genome 3.1 (Rnor6 Ensembl release 6.0 - https://www.ensembl.org/Rattus_norvegicus/Info/Index). Subsequently, DESeq2 package version 3.12 (http://bioconductor.org/packages/release/bioc/html/DESeq2.html, RRID:SCR_015687) (Love et al., 2014) was used to calculate differentially expressed genes (DEGs) between subfields and carry statistical analysis. DESeq2 uses the median of ratios method to normalize the raw counts represented by the number of reads previously aligning to each gene (Love et al., 2014). This method fixes the raw counts for library size and is compatible with large numbers of DEGS. Next, DEGs were submitted to enrichment analysis using clusterProfiler package (https://bioconductor.org/packages/release/bioc/html/clusterProfiler.html, RRID:SCR_016884)) (Yu et al., 2012) and the functional profiles were classified into KEGG Pathways and GO Terms - Biological Processes (BP). Differential gene expression was considered significa when adjusted p-value< .05. For all enrichment analyses, terms and pathways were considered significantly different when p < .05 (after adjustment for multiple comparisons - Bonferroni Test).

### 2.4 Genome wide comparison to mouse data for cross-validation

In order to cross-validate our RNA-seq results, in the present study we explore mice orthologous genes from previous mice hippocampus RNAseq studies. Previous RNA-Seq data from rats and mice tissues already demonstrates a similarity cluster across species (Söllner et al., 2017). The majority of orthologous genes have high sequence conservation and high correlation coefficients (Söllner et al., 2017). We analyzed the Cembrowski et al., 2016 published mice dataset (GSE74985) to directly compare and examine the reproducibility of our rat hippocampal functional analysis and potential markers. Mouse data was downloaded for CA2, and for CA1, CA3 and DG, reads from dorsal and ventral areas were combined for each subregion. For example dorsal CA1 and ventral CA1 reads were combined for a total CA1 data. Reads were aligned using the StarAligner 2.6 program with the *Mus musculus* genome (GRCm38) and subjected to the same pipeline described above using DESeq2 and clusterProfiler. DEGs and functional analysis results were identified and compared to find similarities and divergences across species.

## 3 Results

### 3.1 Identification of DEGs and samples visualization

**Fig. 1:**
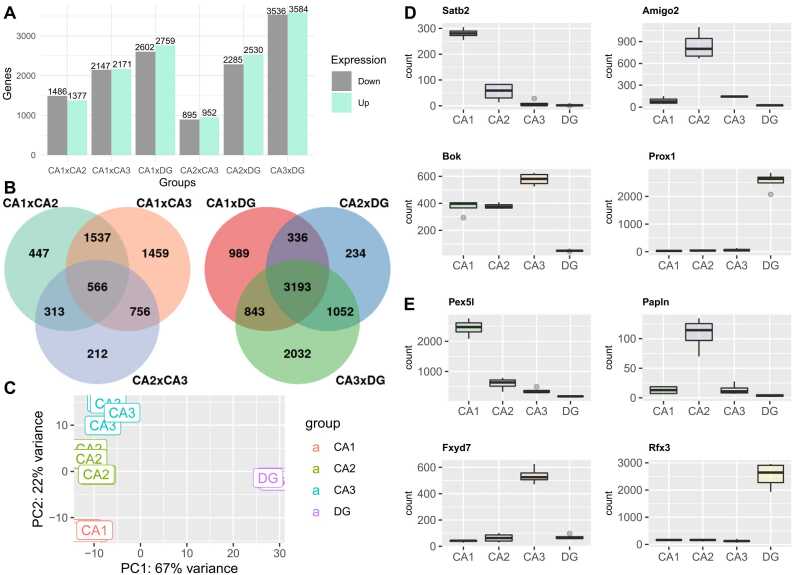
Intragroup and intergroup variability **(A)** Barplot of the transcriptomics data displaying the number of differentially expressed transcripts identified in each hippocampal subfield comparison. **(B)** Venn diagram representing common and unique differentially expressed genes in all comparisons. **(C)** PCA graphic for hippocampal gene expression data displaying an evident cluster of samples on the different regions of the hippocampus. **(D)** Plotcounts of distinct marker genes for each hippocampal subfield.

We ran six pairwise comparisons in DESEQ2 (low counts filter > 10), comparing all subfields between themselves, and obtained the following DEGs results (adjusted p < 0,05) 2863 (CA1vsCA2), 4318 (CA1vsCA3), 1847 (CA2vsCA3), 5361 (CA1vsDG), 4815 (CA2vsDG) and 7120(CA3vsDG). For a complete list of all DEGs refer to Supplementary Table 1. The quantities of DEGs are represented in Fig. (1A). All uniquely and commonly DEGs are represented in the Venn diagram (Fig. 1B). PCA displays an evident clustering of samples of the different regions of the hippocampus: CA1, CA2, CA3, and DG (Fig. 1C).

### 3.2 Molecular markers for hippocampal subfields

Known molecular markers such as ‘*Satb2*’, ‘*Amigo2*’, ‘*Bok*’ and ‘*Prox1*’, for subfields CA1, CA2, CA3, and DG respectively (Hamilton et al., 2017), presented unique expression for those subfields in our dataset (Fig. 1D). In addition, we evaluated potential new marker genes among the DEGs in the dataset unique to each subfield (Fig. 1E). The large number of DEGs allows us to delimit 4 fold change or greater as differential expression cut-off and facilitates the observation of contrastant gene expression comparing a subfield against all others. For the whole set of potential marker genes, please refer to Supplementary Table 2.

### 3.3 Pyramidal to pyramidal subfiled comparisons

#### 3.3.1 CA1vsCA2

**Fig. 2:**
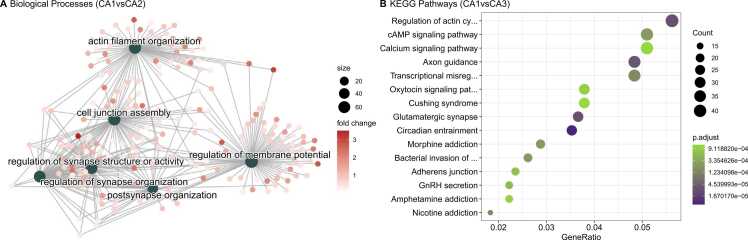
Top significant biological processes of CA1vsCA2 and KEGG pathway of CA1vsCA3 differentially expressed genes **(A)** A net plot of top enriched biological processes from GO analysis of abundant CA1 genes (CA1vsCA2). **(B)** A dot plot of top enriched KEGG pathways of abundant CA1 genes (CA1vsCA2). All enriched pathways and biological processes displayed reached adj.p-value< .05. For the whole set of enriched biological processes and pathways, please refer to Supplementary Table 3 and 4.

**Fig. 3:**
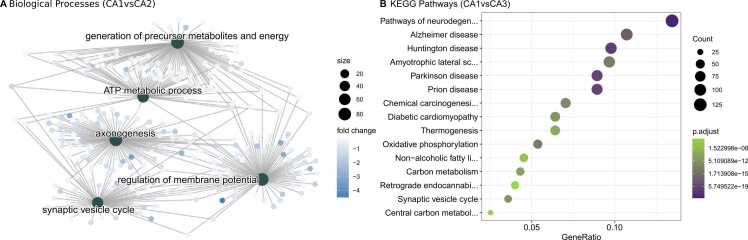
Top significant biological processes of CA1vsCA2 and KEGG pathway of CA1vsCA3 differentially expressed genes **(A)** A net plot of five enriched biological processes from GO analysis of abundant CA2 genes (CA1vsCA2). **(C)** A dot plot of top enriched KEGG pathways of abundant CA3 genes (CA1vsCA3). All enriched pathways and biological processes displayed reached adj.p-value< .05. For the whole set of enriched biological process and pathways, please refer to Supplementary Table 3 and 4.

**Fig. 4:**
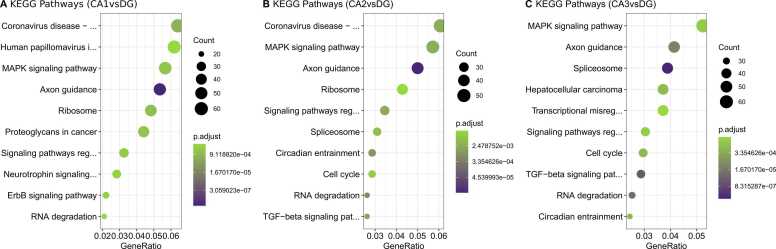
Top significant KEGG pathway of most abundant genes in DG **(A)** A dot plot of top enriched KEGG pathways of abundant DG genes (CA1vsDG). **(B)** A dot plot of top enriched KEGG pathways of abundant DG genes (CA2vsDG). **(C)** A dot plot of top enriched KEGG pathways of abundant DG genes (CA1vsDG). All enriched pathways and biological processes displayed reached adj.p-value< .05. For the whole set of enriched biological process and pathways, please refer to Supplementary Table 3 and 4.

GO and KEGG analysis revealed a distribution of 111 significant (p.adjust < 0.05) BPs and 126 significant (p.adjust < 0.05) pathways for CA1vsCA2 considering all DEGs (See Supplementary Table 3 and 4). Splitting DEGs into log2FoldChange > 0 and log2FoldChange < 0 genes lists and using CA1 as reference evidenced other enriched terms. The top list of enriched GO are demonstrated in Fig. 2A (more abundant in CA1) and Fig. 3A (more abundant in CA2). Fig. 4.

#### 3.3.2 CA1vsCA3

We found 118 BPs and 145 pathways significantly enriched (p.adjust <0.05) for CA1vsCA3′s GO and KEGG analysis (See Supplementary Table 3 and 4). The top list of enriched pathways are demonstrated in Fig. 2B (more abundant in CA1) and Fig. 3B (more abundant in CA3).

#### 3.3.3 CA2vsCA3

A total of 110 BPs and 60 pathways significantly enriched (p.adjust <0.05) were found for CA2vsCA3′s GO and KEGG analysis (See Supplementary Table 3 and 4). We also did the analysis of solemnly log2FoldChange > 0 and solemnly log2FoldChange < 0 gene lists separately using CA2 as reference to obtain more specific terms about the differences between the subfields and to improve the search for terms.

### 3.4 Granular to pyramidal subfield comparisons

GO analysis revealed the following distribution: 128 significantly enriched BP (p.adjust <0.05) for CA1vsDG; 133 significantly enriched BP (p.adjust <0.05) for CA2vsDG; and 102 significantly enriched BP (p.adjust <0.05) for CA3vsDG (See Supplementary Table 3). We also did the KEGG pathway analysis of the most abundant genes using DG as reference (Fig. 3A-C). For a complete list of KEGG pathways refer to Supplementary Tables 4.

### 3.5 Cross-validated functional analysis and potential marker genes

For genome wide comparison we analyzed the published mouse data (Cembrowski et al., 2016) and our rat data independently at the sample level by principal component analysis. The results from PCAs show a consistent clustering of the samples by subfield in both species (Fig. S2). In both datasets granule neurons from DG are distant from CA's subfields, while pyramidal neurons are clearly segregated in CA1, CA2 and CA3 subfields.

For the downloaded mice dataset we obtained the following number of DEGs (adjusted p < 0,05) for the pairwise comparisons: 4861 (CA1vsCA2), 3470 (CA1vsCA3), 3833 (CA2vsCA3), 6954 (CA1vsDG), 8528 (CA2vsDG) and 7192 (CA3vsDG). Next, we found the following commons orthologous DEGs between rat and mice pairwise groups: 1316 (CA1vsCA2), 1452 (CA1vsCA3), 806 (CA2vsCA3), 2604 (CA1vsDG), 2740 (CA2vsDG) and 3282 (CA3vsDG). Furthermore, we identified the following number of common functional analysis results between rat and mice pairwise comparisons (KEGG pathways): 98 (CA1vsCA2), 92 (CA1vsCA3), 56 (CA2vsCA3), 115 (CA1vsDG), 109 (CA2vsDG) and 115 (CA3vsDG). For a complete list of all DEGs, common orthologous DEGs and common functional analysis terms refer to Supplementary Table 5, 6 and 7. Then, we plotted the top enriched KEGG terms of all mice subfield comparisons to cross-validated the common terms found in rat results (Fig. S3).

Additionally, we compared the orthologous potential marker genes between the datasets in the context of 4 fold change or greater as differential expression cut-off. We found a high number of exclusively and similar potential marker genes to both species for all subfields (Fig. S4). For the total comparison between rat and mice potential marker genes refer to Supplementary Table 7.

## 4 Discussion

In the present study we used the LCM technique coupled with RNA-seq to identify genes that are differentially expressed when comparing different subfields of the rat hippocampus. Transcriptomic studies of the brain using RNA-sequencing techniques such as single-cell and single-nucleus RNA-seq have the great advantage of extensively identifying cellular subpopulations expression patterns. However, such techniques may not properly characterize RNAs that are present outside the nucleus due to dendritic or cell body compartment loss during nucleus isolation (Lacar et al., 2016; Armand et al., 2021). Thus, LCM/RNA-seq may still be a favorable option for an accurate tissue collection that allows profiling the transcriptome of a morphologically identifiable neuronal population. Such approach has the advantage of including in the transcriptome analysis RNAs present in cellular compartments outside the cell nucleus, such as dendritic and periaxonal regions.

We aimed to separately analyze CA1, CA2, CA3 pyramidal layers, and DG granular layer to contrast the transcriptional profile of these neuronal populations. Previous studies have already described differences and similarities between pyramidal and granular cells (Cembrowski et al., 2016, Greene et al., 2009, Lein et al., 2004) but the present work is the first to employ the accuracy of LCM for micro-region delimitation associated with RNA-seq for pairwise comparison of the rat hippocampus subfields and to quantify the differences in expression levels. Results obtained in this study provides data that may allow a deeper understanding of the rat hippocampus based on biological processes terms and quantitative transcriptional differences.

We found that the comparison that has the largest number of DEGs is CA3vsDG (Fig. 1A). In addition, the PCA emphasizes the greatest variability between CA3 and DG in both PC1 and PC2 axes (Fig. 1B). Interestingly, 3193 out of 7120 DEGs in CA3vsDG overlap in CA2vsDG and CA1vsDG comparisons (Fig. 1C), which shows how pyramidal and granule neurons have well-defined contrasting expression patterns. We also found that CA1vsCA3 has the largest number of genes differentially expressed in the pyramidal comparison. On the other hand, CA2vsCA3 shows 1847 DEGs, being more similar than any other comparisons, which is consistent with other CA2 transcriptomic data (Farris et al., 2019). These subfield gene variations were already expected due to the role of anatomical differences, connections, firing properties (Kesner and Rolls, 2015, Mizuseki et al., 2012), and distinctive gene profiles on the hippocampus that each subfield has (Cembrowski et al., 2016). In summary, our data suggest that CA3 neuronal expression profiles are remarkably more distinct to CA1 and DG, however, CA3 shares more similarity to CA2.

In order to identify potential hippocampal cell population markers, we searched for genes with an expression four times higher (>4 fold change) when comparing a subfield to all others. Notably, although some of our identified marker genes were previously described as markers in the literature, many of the discovered marker genes were novel for the rat hippocampus (Fig. 4 D). In an example of potential marker genes, we have identified the gene *Pex5l* (peroxisomal biogenesis factor 5-like) for CA1, which is crucial to the establishment of a dendritic gradient of HCN1 channels and contributes to hyperpolarization in these neurons (Piskorowski et al., 2011). In another example, we also found *Rfx3* (regulatory factor X3) with higher expression in DG, which could directly regulate *Fgf1* (fibroblast growth factor 1) (Hsu et al., 2012) and contribute to DG neurogenesis (Ma et al., 2009). Therefore, our data indicates a great number of potential novel marker genes for each of the rat hippocampal subfields.

We compared these genes against previously known markers from (Cembrowski et al., 2016) and found similarities and divergences between rat and mice hippocampus (see Supplementary Table 5). For instance, our dataset has not identificated *Maob* (monoamine oxidase B) and *Scgn* (secretagogin, EF-hand calcium binding protein) as a CA2 marker gene or *Fybcd1* (fibrinogen C domain containing 1) as CA1 marker gene but, like (Cembrowski et al., 2016), we have identificated *Wfs1* (wolframin ER transmembrane glycoprotein) and *Fgf2* (fibroblast growth factor 2) as potential markers for CA1 and CA2 respectively. Intriguingly, recent studies have shown different protein expression patterns in the mouse and the rat hippocampus (Münster-Wandowski et al., 2017, Radic et al., 2017). Our dataset not only recapitulated some of such differences, but also revealed unidentified genes that may be directly correlated with the differences between rat and mouse hippocampus.

Next, we focused on the ontology analysis to characterize the profile of all DEGs in the pyramidal comparisons. For the discussion on ontology analysis we chose to discuss genes that are essential for the biological processes or biochemical pathways being considered and genes that are more thoroughly functionally characterized in the literature. Our analysis indicates that the majority of significant DEGs are enriched for ‘*ATP metabolic processes*’ and ‘*generation of precursor metabolites and energy*’ for CA2 and CA3 subfields when compared to all other regions. These findings are consistent with our cross-validated mice results (Figure S3) and other studies also point to more transcripts related to energetic processes, like glycolysis enzymes in the CA3 subfield (Datson et al., 2004, Greene et al., 2009). Our findings indicate that genes typically related to glycolysis, oxidative phosphorylation, gluconeogenesis, and lipid catabolic process are more abundant in CA2 and CA3 subfields.

We also found that CA2 and CA3 neurons have a higher level of expression of genes responsible for the regulation of oxidative stress such as *Atox1* (antioxidant 1 copper chaperone), *Sod1/Sod2* (superoxide dismutase 1 and 2), *Aldh1a1* (aldehyde dehydrogenase 1 family member A1), and all thioredoxin reductases. Our results were similar to those of Yin et al. (2017), which shows the greater reducing capability of CA3 than CA1 due to higher expression of thioredoxin reductases. We also hypothesized that the same may occur in the CA2 subfield since they share many similarities to the CA3 subfield. Interestingly, CA1 neurons are more sensitive than CA3 neurons to oxidative stress (Lana et al., 2020) and a low expression of oxidative stress regulators genes may be responsible for the lack of resistance of CA1 neurons to the damage caused by any inhibition of mitochondrial activity in these cells. In our study, CA1 pyramidal neurons have shown a lower expression of metabolism related genes when compared to other subfields. Furthermore, the analysis revealed that synaptic vesicle cycle genes, such as *Syp* (synaptophysin), *Vamp1* (vesicle-associated membrane protein), and *Syt11* (synaptotagmin 11) are more abundant in CA3 and CA2 and may correlate to intense energy metabolism and capability of greater synaptic vesicle release and/or recycling in these neurons (Pathak et al., 2015, Yuan et al., 2018). Similar results for CA3 mice data were found also enriched for synapse vesicle terms (Fig. S3). Here we report that CA2 and CA3 pyramidal neurons express more genes responsible for energetic processes, regulation of oxidative stress, and synaptic vesicle cycle.

Actin regulation is a crucial part of neural development for the growth of dendrites and axons. Our analysis indicates more transcripts related to ‘*synapse organization*’ and ‘*actin filament organization*’ in CA1. These results concur with mice CA1 data comparison (Fig. S3). We found actin cytoskeleton signal transducers *Rnd1* and *Rnd3* (Rho GTPases 1 and 3) more abundant in CA1, implicating in a possible higher regulation of neuronal morphogenesis in this subfield (Luo, 2000). In addition, we also found important genes involved in neurite formation like *Map2* (microtubule-associated protein 2), *Twf2* (twinfilin actin-binding protein 2), and *Tpm1* (tropomyosin 1) (Gray et al., 2017; Leif Dehmelt, 2005; Yamada et al., 2007). Furthermore, we found that the transcription factor *Srf* (serum response factor), a classical regulator of several cytoskeletal genes, has a higher expression CA1 when compared to other subfields. *Srf* is critical to induce a gene expression pattern involved in the maintenance of long-term potentiation (LTP), since CA1 pyramidal neurons missing *Srf* exhibit an attenuation of both the early and late phases of LTP (Ramanan et al., 2005). Moreover, we found in CA1 a higher expression of *Myosin Vb*, a protein that has been established as a functional connection to recycling endosomes (Hammer & Sellers, 2011). Interestingly, recycling endosomes contribute to the regulation of AMPA receptors to the plasma membrane (Park et al., 2004) and in the present dataset the CA1 subfield has a higher expression of all four AMPA subunits(*Gria1, Gria2, Gria3, Gria4*) than CA2, CA3, and DG. Spine enlargement dependent on actin increases AMPA-receptor exposure in the membrane, further modulating LTP (Matsuzaki et al., 2004). Thus, the CA1 subfield expression of more actin regulatory genes may be related to LTP regulation and AMPA receptors.

CA2 has remarkable characteristics that differ from other CA subfields, like the lack of LTP and reduced synaptic plasticity (Zhao et al., 2007). However, in our analysis, CA2 was also enriched for ‘*actin filament organization*’ and ‘*synapse organization*’ genes in the CA2vsCA3 comparisons. Therefore, the lack of synaptic plasticity in CA2 may not be explained by a lack of expression of genes associated with these functions. Compared to CA1 and CA3, CA2 neurons are known to be more resistant to trauma, seizure activity, and ischaemic insult, but the mechanisms responsible for such differences are not yet fully characterized (Dudek et al., 2016). Among the transcripts involved in cell survival and apoptosis, we identified that CA2 has a higher expression level of *Atp8a1* (ATPase phospholipid transporter 8A1), *Tp73* (tumor protein p73), *Nes* (Nestin), and *Cd74*. The lack of *Atp8a1* in hippocampal neurons is related to externalization of phosphatidylserine (Levano et al., 2012), which is associated with apoptosis (Fadok et al., 2000). Higher expression of *Atp8a1* in CA2 neurons may reduce phosphatidylserine externalization and subsequent programmed cell death, offering a mechanism of neuroprotection. Furthermore, one critical component of the development, maintenance, and survival of neurons is the *p73* pathway, which may also play a role in CA2, since it has a higher expression in this subfield. Deletion of *Tp73* isoforms results in hippocampal dysgenesis and an impaired organization of CA1 and CA3 subfields (Yang et al., 2000). In addition, our data also show a higher expression of calcium-regulating proteins in CA2, such as *Casr* (calcium-sensing receptor), *Trpc3* (transient receptor potential cation channel C3), *Cacng5* (calcium voltage-gated channel auxiliary subunit gamma 5), *Cacna2d3* (calcium voltage-gated channel auxiliary subunit alpha2delta 3), and *RGS14* (regulator of G protein signaling 14), a classical modulator of calcium signaling and LTP suppression in this hippocampal subfield (Evans et al., 2018, Lee et al., 2010). Similar findings are represented in other studies and support the idea that the lack of LTP is related to CA2 neurons expressing a large number of calcium-regulating proteins (Simons et al., 2009). Therefore, these transcripts may present neuroprotective effects and play a role in intracellular signaling in this cell population.

A higher expression of protein synthesis genes in DG granule cells is well-known and was described in other studies (Datson et al., 2004, Greene et al., 2009). In our data, enriched BP terms for DG most expressed genes were mostly related to the ‘*ribosome*’, ‘*RNA degradation*’, and ‘*spliceosome*’ and we found a large number of ribosomal proteins in DG, such as *Rpl3*, *Rpl4*, *Rpl5*, *Rpl7*, *Rps15*, *Rps16*, *Rps24* and *Rps28*. The analyzed mouse data confirmed enriched BP terms like ‘ribosome’ and ‘spliceosome’ for DG (Figure S3). A higher expression level in the DG of genes involved in ribosomal biogenesis may indicate a robust ribosomal apparatus supporting dendrites’ growth and maintenance (Slomnicki et al., 2016). The DG subfield is capable of neurogenesis throughout life (Ninkovic et al., 2007) and protein synthesis may play a role in proliferation/differentiation and contribute to neuronal turnover in this region. In addition, we confirm the higher expression of pluripotency and neurogenesis molecular biomarkers like *Prox1* (prospero homeobox 1), *FoxO3* (forkhead box O3), *Calb1* (calbindin 1), and *Gfap* (glial fibrillary acidic protein) (Zhang and Jiao, 2015). We also found a higher expression of neurotrophins like *Bdnf* (brain-derived neurotrophic factor) and *Nt-3* (neurotrophin-3), essential molecular mediators to stimulation of protein synthesis and synaptic plasticity in the nervous system (Aakalu et al., 2001). Furthermore, our transcriptomic analysis shows an abundance of alternative splicing factors in DG. Alternative splicing is an important mechanism that regulates the transcript isoforms and generates protein diversity in the cell (Su et al., 2018). We found an up-regulation of *Srsf1* (serine and arginine rich splicing factor 1), *Srsf2* (serine and arginine rich splicing factor 2), *Srsf3* (serine and arginine rich splicing factor 3), *hnRNP* U (heterogeneous nuclear ribonucleoprotein U), *hnRNPa1* (heterogeneous nuclear ribonucleoprotein A1), and *Rbfox1* (RNA binding fox-1 homolog 1) that suggest a greater regulatory mechanism for mRNA isoforms. This study is consistent with previous findings that protein synthesis is increased in the granular neurons of DG and sheds light that splicing factors are enhanced in DG, however future studies are still needed to detect splicing events in the hippocampal subfields.

The transcriptome data generated in the present study uncover the functional profiles and potential molecular mechanisms that underlie the distinction in CA1, CA2, CA3, and DG of rat hippocampal subfields. The gene ontologies enriched in this dataset reveal multiple biological processes such as actin regulation genes and AMPA receptors for CA1; several metabolic processes for CA2 and CA3 transcripts, and ribosome/spliceosome for DG. Many biological processes found enriched in the present dataset have been previously associated with specific characteristics of each hippocampal subfield, however here we present an extensive list of molecular components for each of these processes. Furthermore, the RNA-Seq approach allowed us to measure precisely the expression levels of transcripts from a large set of genes, revealing unique expressed genes for each subfield, contributing to a number of novel potential markers. The present findings contribute to a deeper understanding of the differences in the molecular machinery expressed by the rat hippocampal neuronal populations, further exploring underlying mechanisms responsible for each subflied specific functions.

## Conflict of Interest

The authors declare they have no conflicts of interest.

## Data Availability

The data that support the findings of this study are openly available in the NCBI GEO Datasets (GSE179101).
