# Supplementary material for: The transcriptome of rat hippocampal subfields
Source: IBRO Neurosci Rep. 2022 Oct 3;13:322–9. doi: 10.1016/j.ibneur.2022.09.009 (PMC9561749; doi:10.1016/j.ibneur.2022.09.009)
Supplement: Supplementary file 1 — Supplementary material [file mmc1.docx]

**Supplementary Information**

**Figure S1 - Nissl-stained sections used for laser microdissection. (A)** Section from dorsal and ventral rat hippocampus. **(B)** Same section from dorsal and ventral rat hippocampus after microdissection.

**Figure S2 - Principal component analysis (PCA) of rat and mouse data (A)** PCA from this paper dataset. **(B)** PCA from Cembrowski data (GSE74985) following the normalization pipeline described in this paper.

**Figure S3 - Bar plot of significant KEGG pathways terms from cross-validated mice data (A)** A bar plot of top enriched KEGG pathways of abundant CA1 genes (CA1vsCA2). **(B)** A bar plot of top enriched KEGG pathways of abundant CA3 genes (CA1vsCA3). **(D)** A bar plot of top enriched KEGG pathways of abundant DG genes (CA1vsDG). **(E)** A bar plot of top enriched KEGG pathways of abundant DG genes (CA2vsDG). **(F)** A bar plot of top enriched KEGG pathways of abundant DG genes (CA3vsDG).

**Figure S4 - Examples of rat and mouse similar possible markers (A)** Plotcounts of CA1 possible markers. **(B)** Plotcounts of CA2 possible markers. **(C)** Plotcounts of CA3 possible markers. **(D)** Plotcounts of DG possible markers. For a complete list of rat and mouse similar possible markers refer to Supplementary Tables 8.

**Supplementary table 1** – List of rat genes differentially expressed in all subfield comparisons.

**Supplementary table 2** – List of possible marker genes with four times more expression (>2 log2FoldChange) in only one rat subfield.

**Supplementary table 3** – List of KEGG pathways significantly enriched based on all, solemnly log2FoldChange > 0 or solemnly log2FoldChange < 0 genes in CA1, CA2, CA3, and DG subfields.

**Supplementary table 4** – List of KEGG pathways significantly enriched based on all, solemnly log2FoldChange > 0 or solemnly log2FoldChange < 0 genes in CA1, CA2, CA3, and DG subfields.

**Supplementary table 5** - List of mice genes differentially expressed in all subfield comparisons.

**Supplementary table 6** - List of common orthologous genes differentially expressed in all subfield comparisons (Rat vs Mouse)
**Supplementary table 7** - List of common KEGG pathways (Rat vs Mouse).

**Supplementary table 8** - List of rat and mouse similar possible markers
