# Supplementary figures and images for: The transcriptome of rat hippocampal subfields
Source: IBRO Neurosci Rep. 2022 Oct 3;13:322–9. doi: 10.1016/j.ibneur.2022.09.009 (PMC9561749; doi:10.1016/j.ibneur.2022.09.009)

A

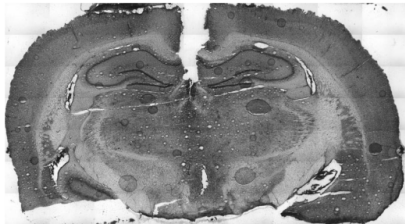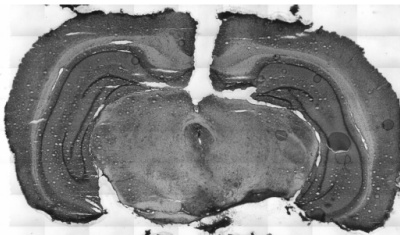

B

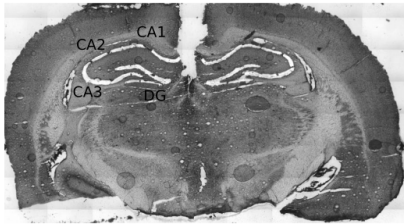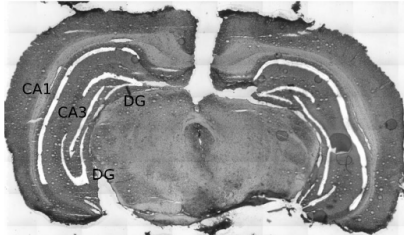

Supplement: Supplementary file 6 — Supplementary material [file mmc2.pdf]

**A** Rat data

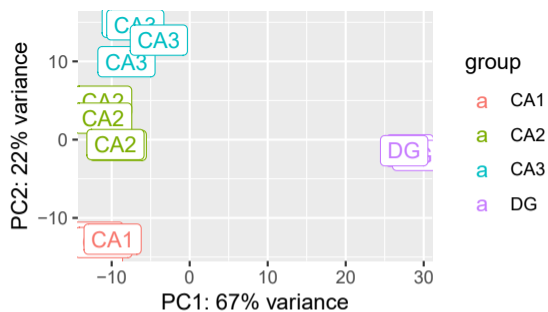

**B** Mouse data

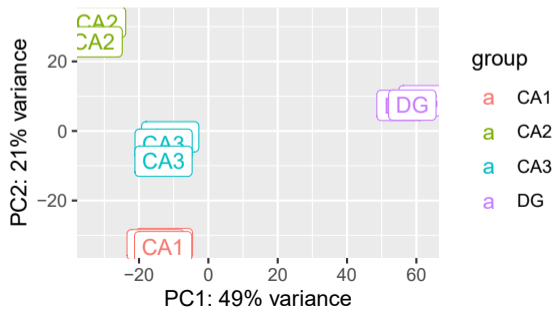

Supplement: Supplementary file 7 — Supplementary material [file mmc3.pdf]
