# Supplementary material for: The transcriptome of rat hippocampal subfields
Source: IBRO Neurosci Rep. 2022 Oct 3;13:322–9. doi: 10.1016/j.ibneur.2022.09.009 (PMC9561749; doi:10.1016/j.ibneur.2022.09.009)
Supplement: Supplementary file 8 — Supplementary material [file mmc4.pdf]

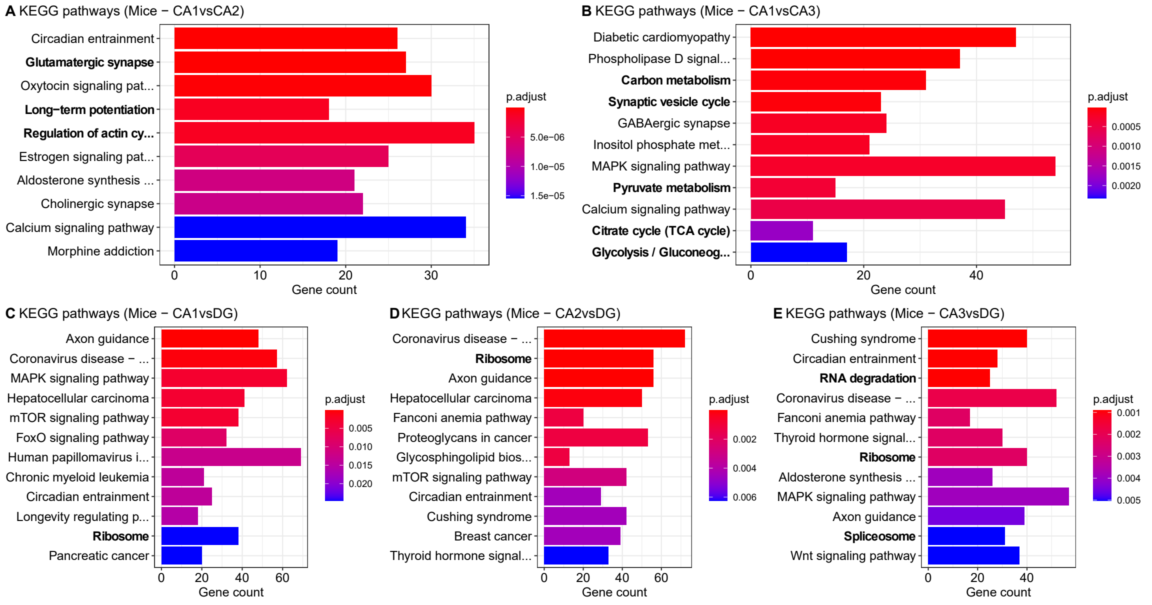
A KEGG pathways (Mice − CA1vsCA2)
Circadian entrainment
Glutamatergic synapse
Oxytocin signaling pat...
Long−term potentiation
Regulation of actin cy...
Estrogen signaling pat...
Aldosterone synthesis ...
Cholinergic synapse
Calcium signaling pathway
Morphine addiction
0 10 20 30
Gene count
p.adjust
5.0e−06
1.0e−05
1.5e−05
B KEGG pathways (Mice − CA1vsCA3)
Diabetic cardiomyopathy
Phospholipase D signal...
Carbon metabolism
Synaptic vesicle cycle
GABAergic synapse
Inositol phosphate met...
MAPK signaling pathway
Pyruvate metabolism
Calcium signaling pathway
Citrate cycle (TCA cycle)
Glycolysis / Gluconeog...
0 20 40
Gene count
p.adjust
0.0005
0.0010
0.0015
0.0020
C KEGG pathways (Mice − CA1vsDG)
Axon guidance
Coronavirus disease − ...
MAPK signaling pathway
Hepatocellular carcinoma
mTOR signaling pathway
FoxO signaling pathway
Human papillomavirus i...
Chronic myeloid leukemia
Circadian entrainment
Longevity regulating p...
Ribosome
Pancreatic cancer
0 20 40 60
Gene count
p.adjust
0.005
0.010
0.015
0.020
D KEGG pathways (Mice − CA2vsDG)
Coronavirus disease − ...
Ribosome
Axon guidance
Hepatocellular carcinoma
Fanconi anemia pathway
Proteoglycans in cancer
Glycosphingolipid bios...
mTOR signaling pathway
Circadian entrainment
Cushing syndrome
Breast cancer
Thyroid hormone signal...
0 20 40 60
Gene count
p.adjust
0.002
0.004
0.006
E KEGG pathways (Mice − CA3vsDG)
Cushing syndrome
Circadian entrainment
RNA degradation
Coronavirus disease − ...
Fanconi anemia pathway
Thyroid hormone signal...
Ribosome
Aldosterone synthesis ...
MAPK signaling pathway
Axon guidance
Spliceosome
Wnt signaling pathway
0 20 40
Gene count
p.adjust
0.001
0.002
0.003
0.004
0.005
