# Supplementary material for: The transcriptome of rat hippocampal subfields
Source: IBRO Neurosci Rep. 2022 Oct 3;13:322–9. doi: 10.1016/j.ibneur.2022.09.009 (PMC9561749; doi:10.1016/j.ibneur.2022.09.009)
Supplement: Supplementary file 9 — Supplementary material [file mmc5.pdf]

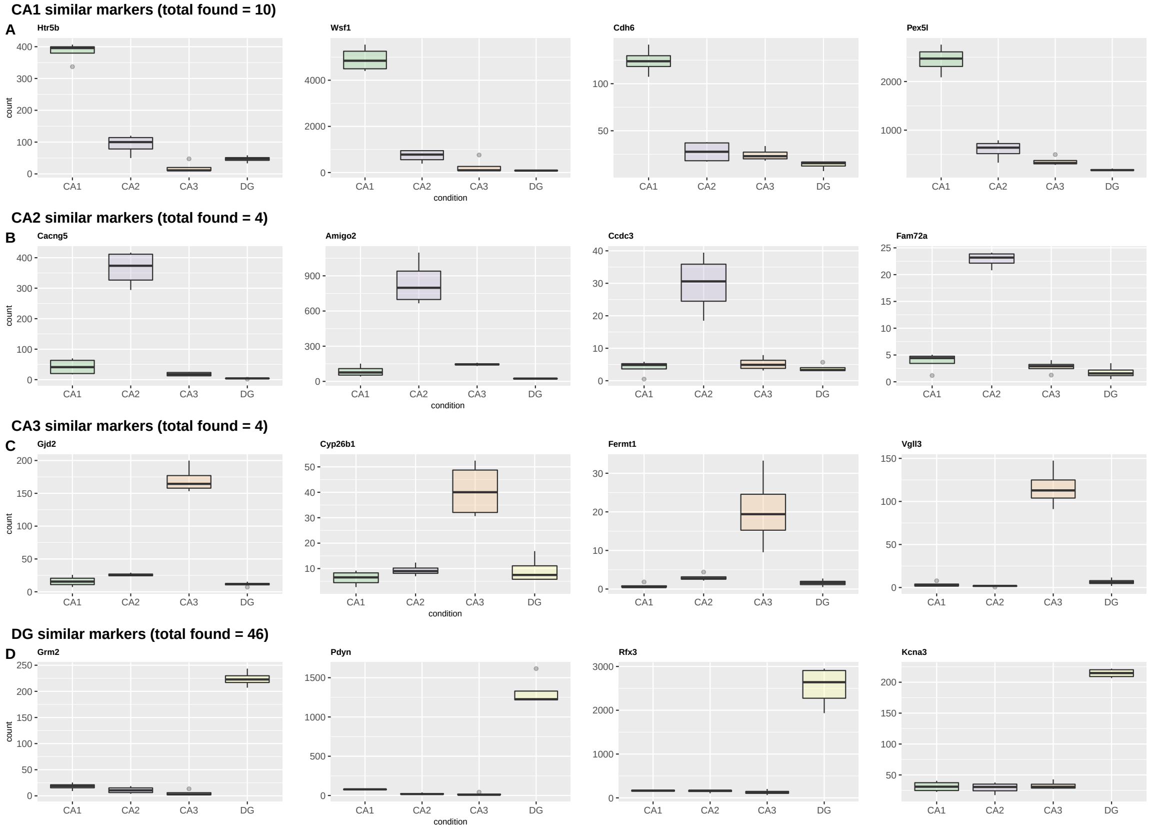
CA1 similar markers (total found = 10)
A
Htr5b
Wsf1
Cdh6
Pex5l
CA2 similar markers (total found = 4)
B
Cacng5
Amigo2
Ccdc3
Fam72a
CA3 similar markers (total found = 4)
C
Gjd2
Cyp26b1
Fermt1
Vgll3
DG similar markers (total found = 46)
D
Grm2
Pdyn
Rfx3
Kcna3
count
condition
CA1
CA2
CA3
DG
